# Stakeholders’ expectations and perceived effects of the pharmacy ownership liberalization reform in Sweden: a qualitative interview study

**DOI:** 10.1186/s12913-016-1637-6

**Published:** 2016-08-12

**Authors:** Kristin Wisell, Ulrika Winblad, Sofia Kälvemark Sporrong

**Affiliations:** 1 Department of Pharmacy, Uppsala University, Box 580, S-751 23 Uppsala, Sweden; 2 Department of Public Health and Caring Services, Uppsala University, Box 564, S-751 22 Uppsala, Sweden; 3 Department of Pharmacy, University of Copenhagen, Universitetsparken 2, 2100 København Ø, Denmark

**Keywords:** Community pharmacy, Regulation, Pharmacy policy, Sweden

## Abstract

**Background:**

Reforms in the health-care sector, including the pharmacy sector, can have different rationales. The Swedish pharmacies were prior to 2009 organized in a state-owned monopoly. In 2009, a liberalization of the ownership took place, in which a majority of the pharmacies were sold to private owners. The rationales for this liberalization changed profoundly during the preparatory work, making it probable that other rationales than the ones first expressed existed. The aim of this study was to explore the underlying rationales (not stated in official documents) for the liberalization in the Swedish pharmacy sector, and also to compare the expectations with the perceived outcomes.

**Methods:**

Semi-structured interviews were conducted with representatives from key stakeholder organizations; i.e., political, patient, and professional organizations. The analysis was performed in steps, and themes were developed in an inductive manner.

**Results:**

One expectation among the political organization participants was that the ownership liberalization would create opportunities for ideas. The competition introduced in the market was supposed to lead to a more diversified pharmacy sector. After the liberalization, the participants in favor of the liberalization were surprised that the pharmacies were so similar.

Among the professional organization participants, one important rationale for the liberalization was to get better use of the pharmacists’ knowledge. However, all the professional, and some of the patient organization participants, thought that the counseling in the pharmacies had deteriorated after the liberalization.

As expected in the interviews, the post-liberalization pharmacy sector consists of more pharmacies. However, an unexpected perceived effect of the liberalization was, among participants from all the stakeholder groups, less access to prescription medicines in the pharmacies.

**Conclusions:**

This study showed that the political organization participants had an ideological basis for their opinion. The political stakeholders did not have a clear view about what the liberalization should lead to, apart from abolishing the monopoly. The perceived effects are quite similar in the different stakeholder groups, and not as positive as were expected.

## Background

Reforms in the health-care sector, including the pharmacy sector, could have different rationales. In some cases, there are obvious problems that need to be solved. Sometimes, the rationales are more ideological. Vogler et al. [1] point out four major reasons for policy changes in the pharmacy sector, i.e.,: 1) changes in the role of pharmacists towards more health-related services. This is in line with the pharmaceutical care movement but is also connected to the expansion of self-care, including more medicines being switched from prescription to non-prescription; 2) economic reasons—e.g., changes in the reimbursement system for pharmacies—either to introduce reimbursement for new services, or diminish the reimbursement; 3) policies related to the overall pharmaceutical system— e.g., a change in which generic versions that are reimbursed; and 4) the amount of regulations is per se seen as a way to pursue certain rationales. One example is that of accessibility. Those in favor of more regulations could argue that increased accessibility would be obtained with more regulations on establishment, since the pharmacies will be more evenly distributed over the country. Those against could argue that less regulation (liberalization) leads to more pharmacies, resulting in increased accessibility.

The pharmacy sector is highly regulated in most countries. This includes ownership, establishment, quality control systems and margins [2]. Pharmaceutical policies, in this case the rules and regulations surrounding the pharmacy sector, have an impact on how, and how well, the sector works, and hence in the long run also on the health of the population. Discussions about and changes in pharmaceutical policy is an ongoing trend in Europe, with changes taking place in many countries [1]. Some of the more comprehensive reforms have included liberalization of ownership of pharmacies in Iceland and Norway (both from systems with pharmacist-owned pharmacies) and most recently in Sweden (from a state-owned monopoly pharmacy chain).

The moves toward privatization can be considered as New Public Management (NPM) reforms. NPM is a term introduced in 1991 by Hood [3], describing a worldwide trend of reforms in the public sector. Reformation has been done in a broad range of areas, including schools and the health-care sector [4, 5]. An important characteristic of NPM is that organizations operated by the state are seen as less effective, making privatization the gold standard [6]. NPM can be seen as a reaction to previous bureaucratic, expensive, and centralized public sector organizations [3]. The competition introduced is expected to lead to more cost-effective organizations, price pressure, and more value for money. Another idea behind NPM is to empower the public service user, by enabling her/him to choose from different providers [7, 8]. Also, when the public organizations are divided and the decisions are made at lower levels, new ideas and innovations are supposed to develop [9].

Prior to 2009, there was a state-owned pharmacy monopoly to sell all medicines in Sweden. All community pharmacies were organized in one pharmacy chain. The liberalization in 2009 removed almost all ownership restrictions, allowing anyone who does not prescribe or produce medicines to own pharmacies. Also, there are no regulations on geographical establishment. Prior to the liberalization reform Sweden had 929 community pharmacies; now the pharmacy sector consists of about 1350 pharmacies. Approximately 165 are run by (mostly single) entrepreneurs, and around 370 pharmacies are still owned by the state [10]. The rest are in large pharmacy chains, owned by private equity firms. From an initial six privately-owned pharmacy chains, there are now three privately-owned chains in the pharmacy sector [11].

Two other reforms were introduced the same year (2009). The first was a reform on non-prescription medicines where specific non-prescription medicines were allowed to be sold outside pharmacies, as long as the owner of the establishment selling them reported this to the authorities [12]. The second was a reform on generic substitution (generic substitution as such already existed). The reform implied that patients had to pay the whole price, if they chose anything other than the cheapest generic medicine [13]. The generics reform was considered necessary in order to get an increase in the number of pharmacies after the liberalization; the money saved on the reform was used to increase the reimbursement to pharmacies [14].

All three reforms were initiated by the center-right government. The funding is still mostly public with additional co-payments from patients.

After the liberalization reforms, governmental agencies and researchers have investigated some of the effects, including consumer satisfaction, costs, and work satisfaction. Some of these investigations show a negative impact on accessibility of medicine and work satisfaction [15–17].

The Swedish pharmacy monopoly was considered to function quite well [18], and there were no obvious complaints from governmental agencies, or from the population. Despite this, the monopoly was abolished in 2009, and a pharmacy sector with almost no ownership regulations was created. The change was more comprehensive and far-reaching than most other pharmacy reforms that were taking place in other European countries at the same time.

The rationales for the ownership liberalization in Sweden were unclear [19]. It seems as though the political rationales [20] for the liberalization were originally economic but that to reduce regulations, was the dominant one in the end [19]. These great changes make it interesting to find out how politicians and other key stakeholders view a liberalization reform in retrospect. How do involved stakeholders, being part of the political process, regard the changes caused by the liberalization?

The aim of this study was to explore the underlying rationales (not necessarily stated in official documents) for the liberalization of ownership in the Swedish pharmacy sector, and also to compare the expectations with the perceived outcomes.

## Method

In order to explore the underlying rationales for the liberalization, a qualitative study using semi structured interviews was used. Qualitative interviews were performed with key stakeholders. The organizations chosen for interviews represented the key actors in the Swedish pharmacy sphere, those which were important in the debate and the formation of the liberalization reform. By asking the group of people at the “inner core” of the liberalization of the ownership regulations in the community pharmacy sector, knowledge about the political process not accessible through studying formal documents can be obtained.

In order to decide which stakeholder organizations to include in the study, written responses from stakeholder organizations on the Swedish Government Official Report were studied, and organizations showing engagement through many or strong opinions were chosen. Also, to further increase the chances of choosing adequate stakeholders, a pharmacy market consultant involved in the process was interviewed before the commencement of data gathering, and the consultant also pointed out organizations that were especially engaged in the process. Later, snowballing (via interviewees) was used to add organizations. This combination of purposeful sampling and snowball sampling [21] increases the probability of obtaining the most adequate sample [22].

The chosen organizations (see Table 1) were contacted per e-mail and, if they agreed to participate, asked to indicate the appropriate person in their organization, typically the most knowledgeable about the liberalization. The participant was then contacted by phone to agree on time and place for the interview. The participants were informed of the aims of the study, and that the interviewer is a licensed pharmacist and a doctoral candidate.

**Table 1:** The organizations included and their attitudes to the liberalization reform before introduction^a^

Stakeholder organizations	Notes	Attitude toward the liberalization of ownership before the reform (according to the Committee Report responses and the interviews)
National Pensioners’ Organization (PRO)	Organization for senior citizens	Negative
The Swedish Association for Senior Citizens (SPF)	Organization for senior citizens	Positive
The Swedish Rheumatism Association (Reumatikerförbundet)	Organization for rheumatism patients	Positive
New Conservatives (Moderaterna)	Governmental party at the time of the reform	Positive
The Liberal Party of Sweden (Folkpartiet)	Governmental party at the time of the reform	Positive
The Centre Party (Centerpartiet)	Governmental party at the time of the reform	Positive
The Christian Democrats (Kristdemokraterna)	Governmental party at the time of the reform	Positive
The Social Democrats (Socialdemokraterna)	Non-governmental political organization (Opposition party at the time of the reform)	Negative
Swedish Association of Local Authorities and Regions (SKL)	Non-governmental political organization (The umbrella organization of the local governments)	Neutral
Swedish Pharmaceutical Society (Apotekarsocieteten)	The goal of the organization is to further pharmaceutical research and to promote high professional standards.	Neutral
The Swedish Pharmaceutical Union (Farmaciförbundet)	Labor union	Negative
The Swedish Pharmacists (Sveriges farmaceuter)	Labor union	Positive

^a^The digit after each quotation does not correspond to the order in which the organizations are presented in Table 1

A semi-structured interview protocol with open-ended questions was constructed based on the study aim and research done on the preparatory work [12]. The interview protocol was modified slightly in regard to the different participants. For example, the organizations were asked to elaborate on their specific expectations of the pharmacy sector, and especially those mentioned in each organization’s consultation responses. These directed questions were used to diminish recall bias. The numbers of questions ranged from 14 to 20, whereof 13 were the same to all participants. To clarify issues, probing questions were also used.

The overall themes in the interviews were the views and rationales for the pharmacy ownership liberalization reform and the perceived effects of the liberalization. Other themes were perceived effects on the health of the population, and the future roles of the community pharmacies and the pharmacist. Questions asked was e.g., what their organization expected from the liberalization of ownership, and their views on how the liberalization had influenced the pharmacies. The interviews were performed face-to-face in a single session, at places chosen by the participants, mostly at their workplaces. Some of the participants had met the interviewer (in other contexts) prior to the commencement of the study. Only the interviewer and the participant were present at the time of the interviews.

The interviews lasted between 35 and 60 min, and were audio-recorded and transcribed verbatim. Field notes were also taken during the interviews. The participants were recruited between February 2013 and September 2013, and interviews were conducted between March 2013 and October 2013.

The interview study was designed by a multi-professional team, consisting of one pharmacist (first author) and two social scientists with long experience of qualitative research (including one political scientist). An interviewer with interviewing experience (first author) conducted all interviews.

The analysis was an inductive content analysis [21, 23] performed in several steps. After four interviews the first and last author read the transcripts and identified tentative themes, first independently, and then in a consensus meeting [23, 24]. This was repeated after eight, ten, and finally after all interviews were done. Modifications of the themes were made after each round. After ten interviews, the interview material was divided into two overall topics: expectations and perceived effects of the liberalization of the Swedish pharmacy sector, and views on the role of community pharmacists. This article deals only with the first topic. Throughout, passages were marked in the transcripts with labels or comments relating to the respective themes. Finally, a meeting was held were all authors discussed the themes and interpretations.

## Results

All organizations asked to participate agreed to do so. The participants are divided into one of three stakeholder groups (see Table 1); political, patient, or professional organization representatives. Altogether, six political, three professional and three patient organization representatives were interviewed, one from each organization; four women and eight men. Quotations have been chosen to illustrate the different opinions. Also, the organizations’ general views on the reform before it took place were analyzed through the Committee Report responses. This is presented in Table 1.

The results section begins with a description of the participants’ (retrospective) expectations of the liberalization reform – first the positive, and then the negative expectations. After that, the perceived outcomes of the liberalization and non-prescription medicines reform are presented – first the anticipated outcomes, and then outcomes that the stakeholders had not foreseen.

The main results are summarized in Table 2.

**Table 2:** Attitudes toward the liberalization reforms before and after the introduction 2009

Expectations and perceived effects	Before the reforms: positive expectations	Before the reforms: negative expectations	After the reforms: positive and neutral outcomes	After the reforms: negative outcomes	After the reforms: negative unexpected outcomes
Accessibility	Better accessibility to pharmacies		Better accessibility to pharmacies		
					Less accessibility to prescription medicines
Ideas	Development of ideas	A lack of development of ideas	A lack of development of ideas		A lack of development of ideas
	Improve specific insufficiencies		Better service and medicine use		
Health effects	More focus on improving the use of medicines				Less focus on improving the use of medicines
					Less connection between community pharmacies and other health care
		Negative health effects and lower increase in number of pharmacies (the non-prescription medicines reform)		Negative health effects (the non-prescription medicines reform)	
Other effects			More focus on commercial goods		

### Before the reforms: positive expectations

The participants’ expectations of positive results included improved pharmacy services through innovations and new ideas, better access through more pharmacies and better use of pharmacists’ knowledge.

Among both participants from the political and the professional organizations in favor of the liberalization, the change of ownership per se was mentioned as a rationale. However, the motives for this stance were not the same. Participants from the political organizations in favor of the ownership liberalization clearly stated that this reform was not done because the pharmacy monopoly had large insufficiencies. Instead, it was performed as a consequence of the premise that government should not own pharmacies. “The reregulation^1^ is done primarily […] not to save money, but in order to create opportunities for ideas, development and to create accessibility” (interview 1). It was also mentioned that there was not a strong public opinion in favor of the liberalization of ownership.

Among participants from the professional organizations, the idea that pharmacists wanted to have the opportunity to own pharmacies was presented. “I think that the feeling of being able to decide by oneself was important” (interview 8). They stated that it was hoped that the liberalization would lead to innovations and a better use of the pharmacists’ knowledge – e.g., better counseling - if pharmacists were allowed to own their own pharmacies. Participants from the political organizations also used this expectation as a rationale for the liberalization; because a professional organization is strongly in favor of the liberalization, this reform must be worthwhile.

### Before the reforms: negative expectations

In the interviews, the major concerns the participants stated they had before the reform, included fear of an oligopoly market without enough competition, and fear of negative health effects because of the sale of non-prescription medicines outside pharmacies.

One fear that was expressed among participants from the political organizations was that the liberalization would not lead to any improvements, as the monopoly was in fact functioning so well. Those representing political organizations in favor of the liberalization brought up the concern that the monopoly might be replaced by an oligopoly. It was stated that in the post-liberalization market it would be almost impossible for small enterprises to survive because of economies of scale and difficulties in creating market segmentation. However, one of the political proponents for the liberalization also feared that the small enterprises might not want to develop new ideas and innovations. “Maybe they just thought: Wow, now we can take over and earn some money” (interview 1).

Participants also expressed that the accessibility goal, defined as an increased number of pharmacies, could be at risk when non-prescription medicines were allowed to be sold outside the pharmacies, and that this would decrease the profitability of the pharmacies. The loss of profit could increase the risk of pharmacies closing down. “We saw disadvantages regarding removing something profitable from the pharmacies” (interview 2).

### After the reforms: positive and neutral outcomes

In this section, the perceived effects (as reported by the participants) of the ownership liberalization and the non-prescription medicines reform are described.

The competition introduced in the market was seen by some of the political organizations as making the pharmacies adjust to the needs of the patients, e.g., adjusting opening hours and improving treatments of patients. “If you have another pharmacy in close proximity, then you try to improve your communication with these patients” (interview 5).

The opinion that the new competition might have had positive effects in the use of medicines was prevalent among the political participants. “A successful pharmacy must make the patients want to return and meet the pharmacists […] And I think that this is a strong driving force for better use of medicines” (interview 4). One of the political organizations stated that starting to sell non-prescription medicines outside pharmacies could have improved public health. “If you measure improved public health by the ability to cure a headache or a stuffy nose quickly, then public health has improved“(interview 6).

An increased focus on offering non-medicines in the pharmacies was mentioned among all stakeholder groups as an effect of the reform. According to one of the governmental political representatives, this was expected: “…a general shift towards sales of more commercial goods in the pharmacies. And that was quite expected” (interview 3).

One effect mentioned by the participants was that smaller pharmacies make stock adaptation harder, which makes accessibility of prescription medicines more difficult than before. Participants from political organizations saw it as a natural consequence that pharmacies – in order to increase in numbers – would be smaller and would therefore not be able to afford big stocks.

### After the reforms: negative outcomes

None of the participants thought that the increased number of pharmacies had improved public health. Different opinions existed regarding both the amount of health-care-related activities, and whether these would affect public health. A presumption was that the increase in pharmacies was not done in order to improve health, but solely to be profitable. Also, among the political organizations, it was mentioned that the perceived increase in pharmacy-based medical tests was done for the”wrong” reason – in order to be profitable, instead of being to increase the health of the population. “It is seldom that we recommend everybody for screening. It has become a purpose of its own” (interview 6).

Even though no questions were asked about the non-prescription medicines reform, many of the participants discussed this reform. Among the patient and professional organizations, possible negative consequences for health were mentioned, as non-prescription medicines are sold at grocery and other stores without the possibility of getting counseling. “We have increased the adverse effects for many [patients]” (interview 11). A few of the political party representatives did not believe sale of non-prescription medicines outside of pharmacies had negative health effects.

### After the reforms: negative unexpected outcomes

The negative unexpected effects, according to the interviewees, included a lack of innovation, and a perceived decrease in accessibility of prescription medicines.

All the participants concluded that the post-liberalization pharmacy market consists of pharmacy chains that are very much alike. The participants were surprised by the lack of specialization/diversity; for example that not many new health-related services had been introduced. Instead the new pharmacies were seen as very much resembling the “old” state-owned pharmacies. There were, however, hopes that a more significant specialization would develop in the future.

One perceived reason for the lack of specialization was that the pharmacies’ low profitability made them focus on “safe” areas. Another explanation expressed among the professional organizations, was that a majority of the new pharmacies belong to big chains: “In that shopping mall there are now two pharmacies […]. There is no reason in the world that people need two” (interview 12).

A third explanation among the participants from political organizations, was a lack of competition, e.g., among the wholesalers.

All the participants from professional-, and some from the patient organizations, thought that counseling in the pharmacies had deteriorated after the liberalization. Reasons given for this included the increased required rate of return (from pharmacy owners), and less focus on professional activities.

Participants from all professional, and from one political organization, considered the liberalization to be negative, as the pharmacists’ accessibility to patients in a pharmacy decreased. “You don’t get the counseling you need” (interview 8).

Some of the participants from patient organizations also viewed the change as negative for their members, because the pharmacists’ ability to focus on pharmaceutical information was reduced. “The focus on profit should not be so overwhelming that you don’t bother to look in FASS [Pharmacopeia Drug Information]” (interview 10). This change in focus was viewed as having affected the use of medicines in a negative direction.

Among the professional organizations’ participants, the move away from professional activities in pharmacies, to more focus on just selling products, was seen as a consequence of the reforms. The change in focus was among all stakeholder groups seen as damaging for the pharmacists’ ability to focus on professional skills.

Participants from all stakeholder groups mentioned that medicines are perceived as less available in the pharmacies after the liberalization. The fact that the pharmacies are smaller, and hence their stocks are also smaller was mentioned among all the participant groups as the main reason for the perceived deterioration. “We don’t have medicines in the pharmacies as much as we had before” (interview 7).

Three main reasons were presented by the participants for this perceived effect. Firstly, the competition introduced in the market was (among a few political representatives) seen as a possible reason, as pharmacies are now more cost-conscious, and therefore have smaller stocks. At the same time, participants from the political organizations were generally surprised that the competition had not led to more pronounced stock adjustment according to patients’ needs. Also, among participants from the political organizations in favor of the liberalization it was stated that the large patient majority had not been affected, but that it is a natural consequence of a market that it is harder for small patient groups to get their medicines. “Some patient groups could be affected negatively” (interview 3).

The other main reason mentioned for the perceived lower accessibility of medicines was that the change in the reimbursement of generic medicines had led to this effect, and that it could induce pharmacies to have smaller stocks. “Next week there will be a new generic […] so maybe you take the chance and have just a small amount” (interview 1).

The third explanation given was that a higher expectation among the patients made them perceive accessibility of medicines as lower.

Lastly, deteriorating collaboration between the community pharmacies and other parts of the health-care sector was mentioned by a political organization participant, who described the connection as less or unchanged since the liberalization.

Also, some of the patient and all of the professional organizations’ participants stated that the collaboration with the rest of the health-care system was lower after the liberalization. “The pharmacies’ role in the health-care system has become weaker” (interview 11). “Nowadays we don’t have time for anything but to dispense” (interview 8).

## Discussion

The perceived effects of the liberalization of ownership were experienced as quite similar by all stakeholder groups, i.e., no differentiation of services, lower accessibility of medicines – but better access to pharmacies, and a commercialization of pharmacies. These effects are in line with those in other liberalized markets [1]. The results show that only a few of the expected effects were considered to be fulfilled, according to the participants; instead, other, unexpected effects were observed. The similarity of opinions is notable as the different stakeholder groups have different ideological as well as practical interests in the pharmacy sector.

To get a development of ideas and diversity was an important underlying rationale for both the political and professional proponents of the reform, as shown in the results. However, all participants from these stakeholders expressed, that the pharmacy sector has not developed in this direction after the liberalization. The two stakeholder groups did not share the same view on *how* the reform could have led to diversity. The participants representing political proponents stated that diversity would be developed through creation of a market, and the professional representatives hoped that it would have happened if only the profession got to own pharmacies. This shows that not only the underlying rationales, but also the mechanism for the change is important to investigate.

### Less accessibility of medicines and less freedom of choice

A common rationale behind NPM-inspired reforms is the introduction of provider choice [25]. However, just getting more pharmacies in the market does not correspond to freedom of choice, because real choice implies a possibility to choose between alternatives that are different. On the contrary, there has even been a diminished opportunity for choice as the change in generic substitution makes it harder for patients to choose anything other than the cheapest drug.

The smaller stocks in the pharmacies were by some participants seen as a natural consequence of the reform, while others were surprised by this consequence of the liberalization. These two effects resulting in less accessibility to medicines (less medicines in stock) and possibly less freedom of choice were not discussed or thoroughly investigated prior to the reform, and can hence be seen as negative *unexpected* effects. The fact that accessibility of medicines was not perceived as a problem prior to the reform is a probable explanation as to why this was not discussed beforehand.

Increased efficiency and price pressure were present as rationales in the beginning of the preparatory work preceding the reform [20]. Despite this, none of the participants mentioned these arguments as a rationale for the reform. This could be explained by the fact that the investigation leading to the reform showed the impossibility of combining more pharmacies and lower public expenses, illustrated in the increase in reimbursement to the pharmacies linked to the liberalization.

The fact that two major rationales were shown to be unfulfilled even before the details of the reforms were decided on did not make the government change its mind. It seems as though they were simply deleted from the agenda, and the change of rules for generic substitution was launched, in order not to obtain *higher* overall costs. The price pressure on generics accomplished not least through the state (via a governmental agency) negotiating prices hence seems to be comprehended as more efficient than a NPM-inspired pharmacy sector.

### Two reforms – one argument

Some participants discussed the reform on non-prescription medicines, even though questions only were asked about the ownership liberalization. It seems as particularly the political representatives confused the ownership liberalization reform with the non-prescription medicines reform; arguments for selling non-prescription medicines outside pharmacies, e.g., accessibility, were used to argue for the liberalization of the ownership. This is interesting, as in reality it is not one reform, or even two reforms that are naturally connected. On the contrary, it would have been possible to liberalize only the sale of non-prescription medicines, or to liberalize only the pharmacy ownership. The former was, for example, the case in Denmark [26].

### Deprofessionalization a result of the liberalization reform

As the results show, both the expected and perceived effects of the ownership liberalization were that the pharmacists’ focus on dispensing and counseling regarding prescription medicines was perceived as diminished, in favor of less professional activities. Two examples of deprofessionalization trends could be seen in the interviews.

Firstly, participants appeared to experience an increased focus on sales of commercial goods in pharmacies. A decreased counseling was highlighted by both the patient and professional organizations as an effect of the reform. The non-prescription medicines reform was seen as one cause for the perceived shift towards more unskilled work tasks; pharmacies have to sell more non-medicines in order to increase profits. If this perception is true this shift might affect the health of the population in a negative direction, as decreased counseling also means that less drug-related problems are detected, and hence medication-related illnesses can increase.

The second deprofessionalization effect reported by participants is that the non-prescription medicines reform could be seen as a loss of a professional domain for pharmacy staff, because non-prescription medicines are now sold in grocery stores, without professional pharmacy staff.

As in Iceland, the Swedish professional organizations differed in their opinions regarding the liberalization reform before it took place [27, 28]. It should be noted that the political organization representatives declared *other* rationales for the reform, compared to those from professional organizations, in favor of liberalization. The former argued that entrepreneurs are important in general, but not pharmacist ownership in particular. The latter argued that non-NPM values, such as better use of the pharmacists’ knowledge, and, as shown in their Committee Report, encouraging professionals, i.e., pharmacists, to own pharmacies were important rationales [29].

The original, openly stated, motives could have been met with other types of policy changes. For instance, non-prescription medicines could have been liberalized for increased accessibility, more pharmacies could have been opened by the state-owned pharmacy company, and drug use services implemented in the same pharmacies, and the changes to reimbursement for generic substitution could have pressured prices further.

Because pharmacies are a part of the health-care sector, it is interesting to note that the political representatives did not consider that the liberalization of ownership could lead to health effects, neither positive nor negative. In the results, this is shown e.g., when a political organization in favor of the reforms is aware that it will lead to a commercialization of the pharmacies, without considering this as a potentially negative effect. It could also be noted that health was not part of the official (governmental documents’) rationale for the reform. Instead some participants from political organizations seemed content with the increased number of pharmacies, but did not reflect on the possible impact on public health – illustrating that pharmacies are not seen as important actors when it comes to public health.

It seems as if liberalization of ownership was wished for, for its own sake, whether the post-liberalization market was more efficient or not. It should be noted that even strong privatization proponents conclude that there is a lack of evidence that privatization reforms lead to greater effectiveness/efficiency [4].

### Limitations

There are some limitations to this study; only one person within each organization was interviewed, and their views might not reflect those of the whole organization. However, most of the participants had been involved throughout the reform, and they were chosen as key participants by their respective organizations. Another limitation is a possible memory bias regarding the questions about the period before the liberalization. The risk of this bias is diminished by involving the consultation responses, i.e., how the organizations positioned themselves in the reform process. These individualized questions also helped the participants to recall the process, which also probably strengthened the validity of the study.

## Conclusion

The perceived effects of the pharmacy sector reform were quite similar in the different stakeholder groups, and the results show that they are generally not as positive after the liberalization as before. It could be argued that the effects of this specific reform should have been more thoroughly investigated, e.g., the effect on the accessibility of prescription medicines in the pharmacies. It is a lesson to be learnt for pharmaceutical policy makers – and others involved – also in other settings: the importance of investigating those aspects that are not the focus of a reform, in order to foresee consequences – desired or not.

However, despite discrepancies between expected and perceived effects, it seems as though no one takes responsibility for the perceived negative effects. Instead, some participants stated that the positive expectations will be fulfilled in the future – without explaining why and how this will happen. There is a need to investigate possible effects before doing this kind of reform, and to include experts that are knowledgeable of the area.

In the interviews, it was clear that some of the participants had limited knowledge about how the pharmacy sector functions. A lesson to be learned is that it is vital that the pharmacy profession is active in questions regarding pharmaceutical and pharmacy policy. In this way, politicians could be helped to work in a more evidence-based way instead of introducing reforms solely based on ideology [30].

## Abbreviation

NPM: New Public Management

## Footnotes

1 In the interview material, different words were used for the ownership liberalization; − i.e., reregulation and deregulation.
